# Longitudinal antibody dynamics after COVID-19 vaccine boosters based on prior infection status and booster doses

**DOI:** 10.1038/s41598-024-55245-9

**Published:** 2024-02-25

**Authors:** Naomi Matsumoto, Ayako Sasaki, Tomoka Kadowaki, Toshiharu Mitsuhashi, Soshi Takao, Takashi Yorifuji

**Affiliations:** 1 Department of Epidemiology, Faculty of Medicine, Dentistry and Pharmaceutical Sciences, Okayama University, 2-5-1 Shikata-cho, Kita-ku, Okayama, 700-8558 Japan; 2 Department of Epidemiology, Graduate School of Medicine, Dentistry and Pharmaceutical Sciences, Okayama University, Okayama, Japan; 3 Center for Innovative Clinical Medicine, Okayama University Hospital, Okayama, Japan

**Keywords:** Viral infection, Epidemiology

## Abstract

Global concern over COVID-19 vaccine distribution disparities highlights the need for strategic booster shots. We explored longitudinal antibody responses post-booster during the Omicron wave in a Japanese cohort, emphasizing prior infection and booster doses. This prospective cohort study included 1763 participants aged 18 years and older with at least three vaccine doses (7376 datapoints). Antibody levels were measured every 2 months. We modeled temporal declines in antibody levels after COVID-19 vaccine boosters according to prior infection status and booster doses using a Bayesian linear mixed-effects interval-censored model, considering age, sex, underlying conditions, and lifestyle. Prior infection enhanced post-booster immunity (posterior median 0.346, 95% credible interval [CrI] 0.335–0.355), maintaining antibody levels (posterior median 0.021; 95% CrI 0.019–0.023) over 1 year, in contrast to uninfected individuals whose levels had waned by 8 months post-vaccination. Each additional booster was correlated with higher baseline antibody levels and slower declines, comparing after the third dose. Female sex, older age, immunosuppressive status, and smoking history were associated with lower baseline post-vaccination antibodies, but not associated with decline rates except for older age in the main model. Prior infection status and tailored, efficient, personalized booster strategies are crucial, considering sex, age, health conditions, and lifestyle.

## Introduction

Since the emergence of SARS-CoV-2 vaccines in northern hemisphere winter 2020, global disparities in vaccine distribution have become a major concern^1–3^. As of May 2023, the majority of the global population has already received their initial vaccine doses (64.4% worldwide and 83.4% in Japan). However, the progress of booster doses after the third dose varies widely by country and region.

Japan widely adopts booster shots (34.5/100 people worldwide and 141.28 boosters/100 people in Japan). Meanwhile, South America lags in booster adoption (initial dose coverage 77.1% and 57.8 boosters/100 people), and Africa is struggling to achieve even sufficient initial vaccination coverage (initial dose coverage 30.6% and 5.7 boosters/100 people)^4^.

The COVID-19 case fatality risk (CFR) stands at 2.0% in regions like Africa and South America with low initial or booster vaccination coverage. In Japan, where booster shots are widespread, the CFR is as low as 0.2%^4^. CFRs are influenced not only by vaccination status but also by each country’s health care infrastructure and hygiene conditions and can therefore not be attributed solely to vaccination rates. Nevertheless, the World Health Organization (WHO) has raised concerns about vaccine distribution disparities^5,6^.

In this context, there are growing calls for clarification of which individuals should receive booster shots^7^. Previous studies report that individuals with prior infection can acquire hybrid immunity through vaccination^8–11^. Indeed, the antibody response after the initial vaccine dose is significantly enhanced in individuals with a prior infection history, resulting in higher peak levels and longer half-lives compared with those who have not had previous infection^12^. However, how the trajectory of antibody levels after vaccination varies with prior infection status has rarely been examined, especially during the epidemic waves involving Omicron variants^13^. Additionally, despite the WHO recommendation for a third (booster) shot^6^, there are limited analyses of post-booster outcomes. Considering the emergence of new SARS-CoV-2 variants, routine booster shots will likely be recommended. However, the clinical importance or utility of repeated (fourth or fifth) booster shots following a third shot has not been assessed^13^. Given the potential for longer antibody half-lives after vaccination in individuals with prior COVID-19 infection, the repeated interval of booster shots may vary according to this history. Such information is crucial in shaping strategies to address the global vaccine distribution disparities.

We conducted a cohort study among residents and workers in a city in Japan, where booster shots are widely adopted, to analyze how longitudinal antibody dynamics after booster shots vary according to prior infection history and booster doses.

## Results

### Participant characteristics

This study included 1763 participants, resulting in a total of 7376 antibody measurements (one to five measurements per participant). The distribution of participant characteristics according to the number of vaccine doses is presented, using both a measurement-based approach (Table 1) and a participant-based approach (Supplementary Table S1 online). The median Immunoglobulin G (IgG) level measured overall was 2963.59 (interquartile range 8674.88), and this increased with the number of administered vaccines. Of the total dataset, 4896 data points, constituting 66.4%, were attributed to women. Measurements from participants in the age group 18–39 years comprised 17.5% of the total, those aged 40–59 years accounted for 43.8%, individuals aged 60–79 years represented 28.4%, and those aged 80 years or above constituted 10.4%. Given the government's strong recommendation for additional vaccinations among older adults and individuals with underlying health conditions, who are considered high-risk, the proportion of older age groups increased with the number of doses administered.

**Table 1:** Descriptive summary by number of vaccine dose administered, based on individual measurements.

	Number of vaccine doses administered			
	3	4	5	All
	(n = 3527)	(n = 2786)	(n = 1063)	(n = 7376)
Sex				
Male	1251 (35.5%)	893 (32.1%)	336 (31.6%)	2480 (33.6%)
Female	2276 (64.5%)	1893 (67.9%)	727 (68.4%)	4896 (66.4%)
Age group (years)				
18–39	874 (24.8%)	345 (12.4%)	72 (6.8%)	1291 (17.5%)
40–59	1854 (52.6%)	1093 (39.2%)	280 (26.3%)	3227 (43.8%)
60–79	597 (16.9%)	960 (34.5%)	535 (50.3%)	2092 (28.4%)
Over 80	202 (5.7%)	388 (13.9%)	176 (16.6%)	766 (10.4%)
Attributes				
General public	1752 (49.7%)	1070 (38.4%)	436 (41.0%)	3258 (44.2%)
Resident or staff at elder facility	696 (19.7%)	820 (29.4%)	320 (30.1%)	1836 (24.9%)
Health care professional	1079 (30.6%)	896 (32.2%)	307 (28.9%)	2282 (30.9%)
Underlying medical conditions				
No	2248 (63.7%)	1550 (55.6%)	557 (52.4%)	4355 (59.0%)
Yes	770 (21.8%)	1018 (36.5%)	474 (44.6%)	2262 (30.7%)
Missing	509 (14.4%)	218 (7.8%)	32 (3.0%)	759 (10.3%)
Immunocompromised or on immunosuppressant medication				
No	2971 (84.2%)	2513 (90.2%)	1013 (95.3%)	6497 (88.1%)
Yes	44 (1.2%)	54 (1.9%)	18 (1.7%)	116 (1.6%)
Missing	512 (14.5%)	219 (7.9%)	32 (3.0%)	763 (10.3%)
Smoking history				
Never-smoker	2408 (68.3%)	2106 (75.6%)	856 (80.5%)	5370 (72.8%)
Smoker	174 (4.9%)	157 (5.6%)	83 (7.8%)	414 (5.6%)
Former smoker	449 (12.7%)	312 (11.2%)	98 (9.2%)	859 (11.6%)
Missing	496 (14.1%)	211 (7.6%)	26 (2.4%)	733 (9.9%)
Alcohol consumption history				
Never-drinker	1885 (53.4%)	1702 (61.1%)	697 (65.6%)	4284 (58.1%)
Drinker	31 (0.9%)	55 (2.0%)	25 (2.4%)	111 (1.5%)
Former drinker	1116 (31.6%)	817 (29.3%)	315 (29.6%)	2248 (30.5%)
Missing	495 (14.0%)	212 (7.6%)	26 (2.4%)	733 (9.9%)
Vaccine type				
BNT162b2	2781 (78.9%)	1329 (48.3%)	3 (0.3%)	4113 (56.1%)
mRNA1273	715 (20.3%)	830 (30.2%)	0 (0.0%)	1545 (21.1%)
Omicron-compatible BNT162b2	25 (0.7%)	571 (20.8%)	1024 (96.3%)	1620 (22.1%)
Omicron-compatible mRNA1273	1 (0.0%)	20 (0.7%)	36 (3.4%)	57 (0.8%)
Other	3 (0.1%)	0 (0.0%)	0 (0.0%)	3 (0.0%)
Days elapsed since vaccination	188.80 (87.19)	78.90 (54.19)	57.08 (31.70)	128.31 (91.06)
History of COVID-19 infection				
No	2914 (82.6%)	2297 (82.4%)	868 (81.7%)	6079 (82.4%)
Yes	613 (17.4%)	489 (17.6%)	195 (18.3%)	1297 (17.6%)
Days elapsed since infection				
No history of infection	2914 (82.6%)	2297 (82.4%)	868 (81.7%)	6079 (82.4%)
0–6 days	2 (0.1%)	0 (0.0%)	1 (0.1%)	3 (0.0%)
7–89 days	311 (8.8%)	235 (8.4%)	97 (9.1%)	643 (8.7%)
90–179 days	173 (4.9%)	121 (4.3%)	39 (3.7%)	333 (4.5%)
Over 180 days	127 (3.6%)	133 (4.8%)	58 (5.5%)	318 (4.3%)
Prevalent strains at infection				
Original strain	18 (1.3%)	20 (2.4%)	7 (3.3%)	45 (1.8%)
Alpha variant	14 (1.0%)	15 (1.8%)	7 (3.3%)	36 (1.5%)
Delta variant	4 (0.3%)	9 (1.1%)	3 (1.4%)	16 (0.7%)
Omicron variant	1360 (97.4%)	800 (94.8%)	198 (92.1%)	2358 (96.0%)
COVID-19 during the study period				
No	2264 (64.2%)	2047 (73.5%)	878 (82.6%)	5189 (70.3%)
Yes	1263 (35.8%)	739 (26.5%)	185 (17.4%)	2187 (29.7%)
IgG median (interquartile range)	1406.12(4418.49)	4465.91(10,465.83)	7939.61(18,567.65)	2963.59(8674.88)

Total of 7376 data points with 1763 individuals.

Data in the table are No. (%).

Among the total data points, 44.2% (3258 data points) were from the general population, 24.9% (1836 data points) corresponded to residents or employees of elder care facilities, and 30.9% (2282 data points) were from health care workers. The most recently administered vaccine type was BNT162b2 (Comirnaty; Pfizer-BioNTech) mRNA vaccine for 5733 data points (total 78.2%: conventional 56.1%, Omicron variant 22.1%) and mRNA-1273 (Spikevax; Moderna) for 1602 data points (total 21.9%: conventional 21.1%, Omicron variant 0.8%). A total of 2262 data points (30.7%) corresponded to participants with underlying health conditions, and 116 data points (1.6%) were in an immunosuppressed state or were using immunosuppressive drugs. With increased number of vaccine doses, the proportion of individuals with no history of smoking or alcohol consumption also increased. Prior COVID-19 infection at the time of measurement was reported for 1297 data points (17.6%).

### Antibody dynamics

**Figure 1:**
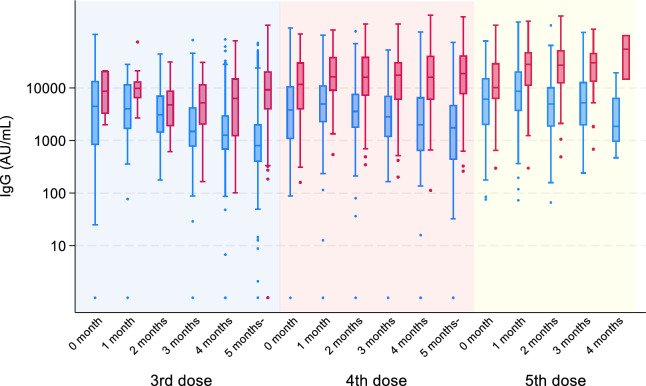
Boxplot of repeated antibody titers by vaccination dose and subsequent days. The blue box with blue dots represents the group never infected with COVID-19, and the red box with red dots represents the previously infected group.

We presented the median, interquartile range, and geometric mean titer of the measured IgG, along with its 95% confidence intervals, stratified by past infection status, the number of most recent vaccinations, and the number of months elapsed since the recent vaccination (Supplementary Table S2 online). We then created box plots illustrating logarithmically transformed IgG values based on the number of doses administered before vaccination and number of months after vaccination (Fig. 1). In the uninfected group, regardless of number of doses administered, antibody levels peaked at 1 month after vaccination and subsequently exhibited a gradual decline over time. This decline rate became less pronounced with an increased number of administered doses. Conversely, the previously infected group displayed minimal declines in antibody levels following vaccination.

### Linear regression analysis

**Figure 2:**
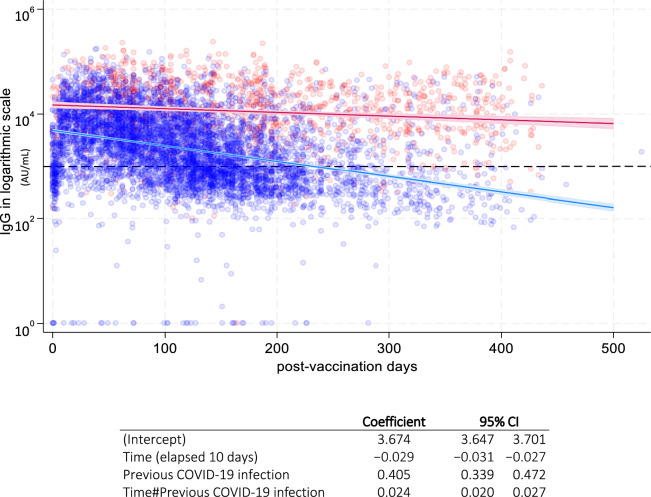
Scatter plot of antibody measurements with overlaid linear regression lines showing temporal changes by prior infection status. Unadjusted (n = 1763 with 7376 data points). The blue line with blue dots represents individuals never infected with COVID-19. The red line with red dots represents the previously infected individuals. The dotted line indicates 1000 AU/mL as a reference. Model details, including intercepts, coefficients, and their corresponding 95% confidence intervals (CIs) are provided in the table below the figure.

In our analysis of antibody dynamics in previously infected and uninfected participants using linear regression, we observed that the previously infected group maintained more sustained antibody levels over time compared with the uninfected group ^13,14^. In uninfected participants, predicted antibody levels fell below 1000 arbitrary units per milliliter (AU/mL) in approximately 220 days; in those previously infected, the predicted antibody levels remained above 1000 AU/mL even after 400 days (Fig. 2).

### Bayesian linear mixed-effects interval-censored analysis

The results of Bayesian linear mixed-effects interval-censored analysis, accounting for uncertainty in measurements exceeding 30,000 AU/mL, are presented in Table 2. Compared with the uninfected group, previously infected participants exhibited higher baseline post-vaccination antibody titers after vaccination (posterior median 0.346, 95% credible interval [CrI] 0.335–0.355), with a gradual temporal decline (posterior median 0.021; 95% CrI 0.019–0.023). Among the infected group, post-infection antibody titers were lower within 7 days of infection (posterior median − 0.317; 95% CrI − 0.324 to − 0.310) and reached their peak within 3 months (posterior median 0.353; 95% CrI 0.344–0.363). With an increased number of vaccine doses beyond the third dose, baseline post-vaccination antibody titers rose (after fourth dose 0.360, 95% CrI 0.344–0.377; after fifth dose 0.464, 95% CrI 0.459–0.472) and the temporal decline became more gradual (after fourth dose 0.009, 95% CI 0.006–0.012; after fifth dose 0.025, 95% CrI 0.014–0.037). With increasing age, baseline post-vaccination antibody titers decreased compared to the age group 18–39 years (age 40–59 years: − 0.020; 95% CrI − 0.036 to − 0.004; 60–79 years: − 0.122; 95% CrI − 0.129 to − 0.114; ≥ 80 years: − 0.254; 95% CrI − 0.273 to − 0.238), and the main model showed the temporal decline of antibody titers. (age 60–79 years: − 0.005; 95% CrI − 0.009 to − 0.000; ≥ 80 years: − 0.009; 95% CrI − 0.016 to − 0.003). Similarly, women exhibited lower baseline post-vaccination antibody titers than men (− 0.122; 95% CrI − 0.132 to − 0.111), but the temporal decline became more gradual (0.008; 95% CrI 0.003–0.016).

**Table 2:** Bayesian linear mixed interval-censored models on antibody titer trends after vaccination (n = 1763 with 7376 data points).

Fixed effects	Posterior median	95% CrI	
(Intercept)	2.751	2.728	2.778
Time (elapsed 10 days)	− 0.019	− 0.028	− 0.010
Previous COVID-19 infection	0.346	0.335	0.355
Previous COVID-19 infection:Time	0.021	0.019	0.023
Post-infection days			
0–6 days	− 0.317	− 0.324	− 0.310
7–89 days	0.353	0.344	0.363
90–179 days	0.283	0.278	0.289
Over 180 days	(omitted)		
Prevalent strains at infection			
Alpha variant	0.645	0.631	0.660
Delta variant	0.015	− 0.006	0.036
Omicron variant	0.631	0.619	0.642
Recent vaccine dose			
4	0.360	0.344	0.377
5	0.464	0.459	0.472
Recent vaccine dose:Time			
4:Time	0.009	0.006	0.012
5:Time	0.025	0.014	0.037
Age group (years)			
Age 40–59	− 0.020	− 0.036	− 0.004
Age 60–79	− 0.122	− 0.129	− 0.114
Over 80	− 0.254	− 0.273	− 0.238
Age group (years):Time			
Age 40–59:Time	− 0.002	− 0.009	0.004
Age 60–79:Time	− 0.005	− 0.009	0.000
Over 80:Time	− 0.009	− 0.016	− 0.003
Female sex	− 0.122	− 0.132	− 0.111
Female sex:Time	0.008	0.003	0.016

Random-effects parameters (unstructured)	Posterior median	95% CrI	
Variance of random intercept	0.178	0.129	0.230
Covariance between random intercept and random slope	− 0.007	− 0.010	− 0.004
Variance of error term for time	0.003	0.003	0.003
Variance of the overall error term	0.190	0.175	0.207

References for the categorical variables are as follows:

No history of previous COVID-19 infection is the reference group for the post-infection days category.

Original strain is the reference group for the prevalent strains at infection category.

Dose of 3 is the reference group for the recent vaccine dose category.

Age < 40 years is the reference group for the age group category.

Male sex is the reference group for female sex.

Time, post-vaccination days (by 10 days), is a continuous variable from days 0 to 500.

The symbol “:” indicates interaction.

CrI, credible interval.

Results from additional analyses, which included a quadratic term for time to assess potential nonlinear trends, Bayesian linear mixed-effects interval-censored models with random-intercept models, and uncensored models with replacement of values above 30,000 AU/ml, showed no substantial differences in outcomes. The Deviance Information Criterion (DIC) was approximately the same for each model (Supplementary Table S3-6 online).

### Supplementary analysis

In the supplementary analysis, excluding 779 data points with missing values for underlying medical conditions, immunosuppressive status, smoking history, and alcohol consumption, we observed that immunosuppressive status and smoking history were linked to a reduction in baseline post-vaccination antibody titers. However, no significant difference was observed in the temporal decline among these factors (see Table 3).

**Table 3:** Bayesian linear mixed interval-censored models adjusting for medical history and lifestyle factors on antibody titer trends after vaccination (n = 1439 with 6597 data points).

Fixed effects	Posterior median	95% CrI	
(Intercept)	2.695	2.634	2.773
Time (elapsed 10 days)	− 0.006	− 0.017	0.001
Previous COVID-19 infection	0.345	0.320	0.365
Previous COVID-19 infection:Time	0.020	0.017	0.023
Post-infection days			
0–6 days	− 0.681	− 0.707	− 0.655
7–89 days	0.379	0.364	0.391
90–179 days	0.270	0.265	0.275
Over 180 days	(omitted)		
Prevalent strains at time of infection			
Alpha	0.618	0.609	0.628
Delta	0.009	− 0.012	0.022
Omicron	0.651	0.629	0.673
Recent vaccine dose			
4	0.396	0.384	0.405
5	0.499	0.490	0.512
Recent vaccine dose:Time			
4:Time	0.005	− 0.002	0.009
5:Time	0.023	0.009	0.039
Age group (years)			
Age 40–59	0.001	− 0.012	0.012
Age 60–79	− 0.109	− 0.124	− 0.090
Over 80	− 0.266	− 0.303	− 0.234
Age group (years):Time			
Age 40–59:Time	− 0.001	− 0.005	0.004
Age 60–79:Time	0.002	− 0.004	0.009
Over 80:Time	0.004	− 0.007	0.015
Female sex	− 0.103	− 0.108	− 0.096
Female sex:Time	− 0.008	− 0.017	0.001
Underlying medical conditions	0.004	− 0.022	0.019
Underlying medical conditions:Time	− 0.002	− 0.011	0.007
Immunocompromised or on immunosuppressant medication	− 0.131	− 0.143	− 0.121
Immunocompromised or on immunosuppressant medication:Time	0.003	− 0.005	0.013
Smoking history			
Smoker	− 0.057	− 0.063	− 0.047
Former smoker	− 0.083	− 0.090	− 0.075
Smoking history:Time			
Smoker:Time	0.003	− 0.003	0.010
Former smoker:Time	− 0.009	− 0.020	0.000
Alcohol consumption history			
Drinker	0.288	0.274	0.298
Former drinker	0.002	− 0.004	0.008
Alcohol consumption history:Time			
Drinker:Time	− 0.024	− 0.049	0.006
Former drinker:Time	− 0.003	− 0.014	0.007

Random-effects parameters (unstructured)	Posterior median	95% CrI	
Variance of random intercept	0.188	0.140	0.243
Covariance between random intercept and random slope	− 0.007	− 0.011	− 0.004
Variance of error term for time	0.003	0.003	0.004
Variance of the overall error term	0.189	0.174	0.206

References for the categorical variables are as follows:

No history of previous COVID-19 infection is the reference group for the post-infection days category.

Original strain is the reference group for the prevalent strains at infection category.

Dose of 3 is the reference group for the recent vaccine dose category.

Age < 40 years is the reference group for the age group category.

Male sex is the reference group for female sex.

No underlying medical conditions is the reference group for the underlying medical conditions category.

Not immunocompromised or on immunosuppressant medication is the reference group for immunocompromised or on immunosuppressant medication category.

Never smoker is the reference group for the smoking history category.

Never drinker is the reference group for the alcohol consumption history category.

Time, post-vaccination days (by 10 days), is a continuous variable from days 0 to 500.

The symbol “:” indicates interaction.

CrI, credible interval.

## Discussion

This study examined the longitudinal dynamics of antibody responses following booster vaccinations during the Omicron variant surge, focusing on prior infection status. Individuals with prior infection exhibited sustained antibody levels after additional vaccinations over time, compared with those who did not have a prior infection. Additionally, as the number of vaccine doses increased, baseline post-vaccination antibody titers increased, with a slower decline observed over time. With increasing age, baseline post-vaccination antibody titers decreased, and the main model showed the temporal decline of antibody titers. Female sex, immunosuppressive status, and smoking history were associated with decreased baseline post-vaccination antibody titers, although no significant differences in decline were observed over time.

In previous studies examining antibody responses after primary vaccination targeting the original strain, individuals with prior infection experienced substantially enhanced antibody responses, leading to elevated peak levels and prolonged half-lives, compared with individuals who did not have prior infection^12^. The same research group reported that antibody responses after three vaccinations during the Omicron epidemic were similarly higher in previously infected individuals, who were more likely to maintain peak antibody titers and longer half-lives^13^. However, some reports have raised concerns about vaccine efficacy, as antibody titers did not increase in previously infected with the Omicron strain than in uninfected after the third dose of vaccination^15^.

Our population-based follow-up study corroborated that individuals with prior infection maintained their antibody levels over time following additional vaccinations, compared with those not previously infected with COVID-19. Previous reports suggest that antibody levels below 1000 binding antibody units per milliliter (BAU/mL) are associated with a significantly increased risk of Omicron-strain infection^13,14^. The unadjusted linear regression performed to visually illustrate the temporal changes in antibody titers between groups in our study also suggested that individuals with prior infection retained antibody levels exceeding 1000 AU/mL, often considered indicative of reduced infection risk, for over 400 days. Bayesian linear mixed-effects interval-censored analysis further demonstrated that an increased number of additional vaccine doses was correlated with rising baseline post-vaccination antibody levels and a milder decline over time. These outcomes suggest the potential impact of additional booster shots to fortify immunity and extend the duration of protection—an essential consideration for nations with ongoing booster programs. In contrast, post-vaccination antibody titers in individuals with no history of prior infection fell below 1000 AU/mL within approximately 8 months. A previous study also reported that longer intervals between BNT162b2 vaccinations may result in higher antibody titers^16^. Given the current global surge in individuals with a history of prior infection^17^ and the ongoing threat of new variants^18,19^, the adoption of annual booster vaccinations may become the prevailing practice^20^.

As factors influencing antibody titer trends, we found that female sex, older age, immunosuppressive states, and smoking history were associated with lower baseline post-vaccination antibody titers, while declines over time were not observed except for older age in the main model. Consequently, earlier booster vaccination, particularly for such vulnerable individuals, may be warranted.

There are several limitations in this study. The study location was limited to one Japanese city, limiting generalizability of the results to the global population. Owing to the absence of N-antibody measurements, there is potential misclassification of infection history. Moreover, we considered antibody levels as a measure of immunity, neglecting other facets of the immune response such as cellular immunity^21–23^. Further long-term follow-up studies are needed to assess the durability of booster shot protection and need for additional booster vaccination.

Our findings yield vital insights into the potential impact of additional vaccine doses on hybrid immunity formation and offer pertinent implications for addressing global vaccine distribution disparities. Prioritization of booster shots for individuals with the greatest need, considering factors such as prior infection and other factors, holds promise for enhancing vaccine promotion strategies in regions with limited vaccine supplies. Further research and international collaboration are essential to effectively combat COVID-19, considering individual- and population-level factors.

## Methods

### Study design and participants

**Figure 3:**
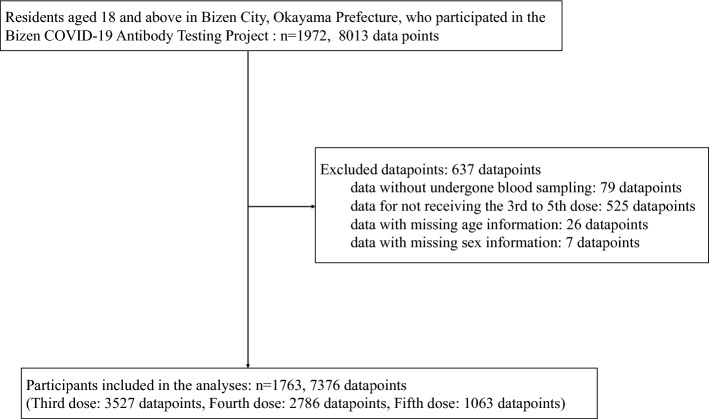
Flowchart of participants.

This prospective cohort study was conducted in a suburban area of Japan, targeting residents and workers aged ≥ 18 years. Bizen City is a small city located in Okayama Prefecture, in western Japan. We recruited 1972 individuals who either held registered residency or were employed in organizations situated in Bizen City. Participation was entirely voluntary. Individuals indicated their interest in study participation by responding to city-wide public announcements, receiving informational leaflets, or after finding information at medical institutions. The recruitment phase spanned from May to June 2022, with data collection between June 3, 2022 and March 27, 2023. During the study period, Japan experienced two major epidemic waves dominated by the Omicron variant, from July to September 2022 and November 2022 to January 2023. Study participants were requested to undergo antibody level measurement approximately every 2 months. Participants were notified about their next antibody measurement appointment via email or telephone, or by the designated contact person within their respective organizations. Participants made an appointment, had their antibody levels measured, and completed a questionnaire survey. Throughout the study, each individual had a maximum of five opportunities for antibody measurement and survey completion. Eligibility criteria included individuals who had received a minimum of three doses of COVID-19 vaccine. Those who never underwent any measurement or lacked information on age or sex were excluded. This study comprised 1763 participants, with a collective total of 7376 antibody measurements taken (ranging from one to five measurements per participant) (Fig. 3).

### Information on COVID-19 vaccination

During the study period, Japan's vaccination strategy was as follows. As of June 2022, initial vaccination and the third booster dose were recommended for all individuals aged ≥ 12 years. Additionally, a fourth booster dose was recommended for individuals aged ≥ 60 years or adults with underlying medical conditions. The required interval between additional doses was at least 5 months. In late July 2022, eligibility for the fourth booster dose was expanded to include health care workers in elder care facilities. Starting from September 20, 2022, all individuals aged ≥ 12 years who had completed their initial vaccination series could receive Omicron-compatible vaccinations. From October onward, the interval for additional doses was adjusted to a minimum of 3 months. This vaccination strategy was aimed to provide comprehensive coverage and adapt to the challenges posed by emerging variants, particularly the Omicron strain, while considering the vaccination needs of specific populations such as older adults and those with underlying health conditions^24^.

Information regarding COVID-19 vaccination among residents of Bizen City, including details such as the number of vaccine doses administered, vaccination dates, and types of vaccines used, was obtained from official vaccination records. For non-residents and individuals lacking official vaccination records in Bizen City, we used self-reported vaccination information, updated at each antibody measurement and survey.

### SARS-CoV-2 antibody levels

SARS-CoV-2 antibody levels were assessed by collecting 30 μL of blood using fingertip sampling with the SARS-CoV-2 IgM & IgG Quantum Dot Immunoassay (Mokobio Biotechnology R&D Center Inc., Rockville, Maryland, USA). This assay specifically targets SARS-CoV-2 spike receptor-binding domain (S-RBD immunoglobulin G [IgG]) antibodies. For samples with limited blood volume, appropriate dilutions were made prior to measurement and subsequent adjustment was made. To assess the temporal decline in antibody levels, antibody titers were logarithmically transformed.

### Information on prior COVID-19 infection

Data regarding prior COVID-19 infection among participants, including information on infection dates, diagnosis dates, and severity of illness throughout the course, were sourced from official prefecture records. Comprehensive recording of COVID-19 infection data in Japan ceased after September 27, 2022. In cases of non-residents and individuals lacking official records in Okayama Prefecture, we used self-reported information on COVID-19 infection, which was updated at each antibody measurement and survey. Based on epidemiological surveys conducted by the National Institute of Infectious Diseases, the prevalent strains at the time of infection were classified as follows: the original strain (before March 2021), the Alpha variant (April 2021–June 2021), the Delta variant (July 2021–December 2021), and the Omicron variant (after January 2022)^25^.

It is important to note that we only considered information about the most recent infection prior to each measurement date in the analyses and did not include future infections occurring after the measurement date.

### Covariates

Information regarding age and sex were collected through the initial survey. In the fifth (final) survey, participants were asked about their height, weight, current medical conditions, immunosuppressive status (including use of immunosuppressive drugs), alcohol history, and smoking history. Those who reported any of the following as current medical conditions were classified as having underlying medical conditions: hypertension, obesity, dyslipidemia, chronic respiratory diseases, chronic kidney disease, diabetes, cardiovascular diseases, cerebrovascular diseases, or malignancies, and body mass index (calculated using height and weight) ≥ 30 kg/m^3^^26^. For individuals who did not respond to the final survey, information regarding underlying medical conditions, immunosuppressive status, alcohol history, and smoking history was unavailable. However, participants who indicated any of the following current medical conditions in the first survey were similarly classified as having underlying medical conditions: hypertension, obesity, dyslipidemia, chronic obstructive pulmonary disease, angina/heart attack, stroke, or malignancy.

### Community collaboration and ethical considerations

This study was conducted with full cooperation from Bizen City, with active participation by its residents and local businesses. Participants were provided with detailed explanations of the research and provided their informed consent before initial measurements. Participants were informed of their right to withdraw from participation at any time during the study. Additionally, as a preventive measure against COVID-19, masks, hand sanitizers, and other items were distributed to participants at each antibody measurement visit. This study adhered to the ethical guidelines for research involving human subjects in the life sciences and medical fields and received approval from the Institutional Review Board of Okayama University Graduate School of Medicine, Dentistry, and Pharmaceutical Sciences (No. K2205-061).

### Statistical analysis

This study targeted participants aged ≥ 18 years who underwent at least one antibody measurement between June 3, 2022, and March 27, 2023 and had received a minimum of three vaccine doses by the time of measurement. After describing the attributes of participants corresponding to each recent vaccination dose, using both a measurement-based and a participant-based approach, we further categorized each IgG measurement value into previously infected and uninfected groups based on the infection status of the participants at the time of measurement. Additionally, we presented the median, interquartile range, and geometric mean titer of the measured IgG, along with its 95% confidence intervals, stratified by past infection status, the number of most recent vaccinations, and the number of months elapsed since the recent vaccination. We then used box plots to depict the logarithmically transformed antibody levels after each vaccination, categorized by the number of doses and time since vaccination.

We used simple linear regression analysis to visually represent the observed data and to qualitatively assess the temporal dynamics of antibody titers across prior infection status. Subsequent inclusion of a quadratic term in the regression model did not match the decay pattern observed in the actual data, as evidenced by the trajectories plotted (Supplementary Fig. S1 online). In particular, the coefficient associated with the quadratic term was insignificantly small, indicating a negligible deviation from linearity. Therefore, we concluded that a linear regression model was more appropriate to illustrate the gradual decline observed in the empirical data over time. It’s important to note that this visualization analysis was designed to elucidate temporal trends and was distinct from our main analysis, which was designed to statistically compare antibody titers between individuals with and without prior infection.

Following the manufacturer’s instructions for the assay kits, considering the uncertainty of measurements exceeding 30,000 antibody units per milliliter (AU/mL), we modeled the temporal decay of antibody levels post-vaccination using a Bayesian linear mixed-effects interval-censored model with noninformative prior distributions^13,27^. We opted for noninformative Jeffreys prior distributions to maintain objectivity in our analysis, especially considering the uncertainty associated with measurements exceeding 30,000 AU/mL. We used a multivariable model, including COVID-19 history (dichotomous), prevalent strains at time of infection (categories: original, alpha, delta, and omicron), days since infection (continuous), the most recent number of vaccine doses (categorical: 3, 4, 5), sex (dichotomous), and age (categories: 10–39, 40–59, 60–79, and ≥ 80 years) as covariates. To assess the temporal decline in antibody levels post-vaccination, interaction terms with time were included for prior infection, vaccine doses, sex, and age. The model incorporated population-level fixed effects, individual-level random effects for intercepts and slopes, and correlations between random effects. The results were upper-censored at 30,000 AU/mL, reflecting the uncertainty of IgG values exceeding the quantification limit. Specifically, data points exceeding 30,000 AU/mL (342/955 datapoints, 26.4% in the previously infected group; 238/5841 datapoints, 3.9% in the uninfected group) were treated as probability distributions that included the upper limit, rather than their actual values.

In sensitivity analyses, we also fitted alternative models, such as the random intercept model and the random intercept and slope model with the quadratic term for time to assess potential non-linear trends. Additionally, the random intercept and slope model without censoring where values above 30,000 AU/ml were replaced, was examined to assess the robustness of our findings. All models were conducted in the Bayesian framework with 2500 burn-in iterations and 10,000 iterations performed in posterior simulation. Model evaluation was performed using the Deviance Information Criterion (DIC). We reported detailed information about the models used in our study, including the rationale and codes, following the Bayesian analysis reporting guideline^28^. Specifically, we used Stata v.18 (StataCorp LLC, College Station, TX, USA) for all analyses and the “bayes: metobit” command to perform the Bayesian multilevel interval-censored analysis, and we included the code for all models in the Supplementary Table S3 online^27,29^.

We also conducted a supplementary analysis including underlying medical conditions, immunosuppressive status, smoking history, and alcohol consumption history as covariates to explore potential additional factors influencing antibody dynamics, and excluding 779 data points in which this information was missing.

### Supplementary Information

Supplementary Information.

## Data Availability

The datasets generated and analyzed during the current study are not publicly available due to privacy and ethical reasons but are available from the corresponding author on reasonable request and approval by Bizen city.
