# Supplementary material for: Longitudinal antibody dynamics after COVID-19 vaccine boosters based on prior infection status and booster doses
Source: Sci Rep. 2024 Feb 25;14:4564. doi: 10.1038/s41598-024-55245-9 (PMC10894855; doi:10.1038/s41598-024-55245-9)
Supplement: Supplementary file 1 — Supplementary Information. [file 41598_2024_55245_MOESM1_ESM.pdf]

S_Table 1. Descriptive summary by number of vaccine dose administered, based on individual participants

	Number of vaccine doses administered			
	3 (n=885)	4 (n=641)	5 (n=237)	All (n=1,763)
Sex				
Male	329 (37.2%)	200 (31.2%)	78 (32.9%)	607 (34.4%)
Female	556 (62.8%)	441 (68.8%)	159 (67.1%)	1,156 (65.6%)
Age group (years)				
18–39	226 (25.5%)	81 (12.6%)	22 (9.3%)	329 (18.7%)
40–59	445 (50.3%)	258 (40.2%)	62 (26.2%)	765 (43.4%)
60–79	154 (17.4%)	216 (33.7%)	117 (49.4%)	487 (27.6%)
Over 80	60 (6.8%)	86 (13.4%)	36 (15.2%)	182 (10.3%)
Attributes				
General public	436 (49.3%)	244 (38.1%)	98 (41.4%)	778 (44.1%)
Resident or staff at elder facility	180 (20.3%)	193 (30.1%)	72 (30.4%)	445 (25.2%)
Health care professional	269 (30.4%)	204 (31.8%)	67 (28.3%)	540 (30.6%)
Underlying medical conditions				
No	482 (54.5%)	343 (53.5%)	128 (54.0%)	953 (54.1%)
Yes	165 (18.6%)	227 (35.4%)	101 (42.6%)	493 (28.0%)
Missing	238 (26.9%)	71 (11.1%)	8 (3.4%)	317 (18.0%)
Immunocompromised or on immunosuppressant medication				
No	636 (71.9%)	558 (87.1%)	224 (94.5%)	1,418 (80.4%)
Yes	8 (0.9%)	12 (1.9%)	5 (2.1%)	25 (1.4%)
Missing	241 (27.2%)	71 (11.1%)	8 (3.4%)	320 (18.2%)
Smoking history				
Never-smoker	518 (58.5%)	466 (72.7%)	188 (79.3%)	1,172 (66.5%)
Smoker	39 (4.4%)	34 (5.3%)	15 (6.3%)	88 (5.0%)
Former smoker	90 (10.2%)	72 (11.2%)	28 (11.8%)	190 (10.8%)
Missing	238 (26.9%)	69 (10.8%)	6 (2.5%)	313 (17.8%)
Alcohol consumption history				
Never-drinker	397 (44.9%)	377 (58.8%)	160 (67.5%)	934 (53.0%)
Drinker	7 (0.8%)	11 (1.7%)	5 (2.1%)	23 (1.3%)
Former drinker	243 (27.5%)	183 (28.5%)	67 (28.3%)	493 (28.0%)
Missing	238 (26.9%)	70 (10.9%)	5 (2.1%)	313 (17.8%)
Vaccine type				
BNT162b2	692 (78.3%)	295 (46.8%)	1 (0.4%)	988 (56.4%)
mRNA1273	177 (20.0%)	197 (31.3%)	0 (0.0%)	374 (21.4%)
Omicron-compatible BNT162b2	13 (1.5%)	133 (21.1%)	227 (95.8%)	373 (21.3%)
Omicron-compatible mRNA1273	1 (0.1%)	5 (0.8%)	9 (3.8%)	15 (0.9%)
Other	1 (0.1%)	0 (0.0%)	0 (0.0%)	1 (0.1%)
Days elapsed since vaccination	180.34 (86.70)	78.89 (54.83)	58.62 (32.62)	127.09 (88.91)
History of COVID-19 infection				
No	742 (83.8%)	521 (81.3%)	193 (81.4%)	1,456 (82.6%)
Yes	143 (16.2%)	120 (18.7%)	44 (18.6%)	307 (17.4%)
Days elapsed since infection				
No history of infection	5 (1.5%)	5 (2.6%)	1 (2.2%)	11 (1.9%)
0–6 days	3 (0.9%)	4 (2.1%)	1 (2.2%)	8 (1.4%)
7–89 days	2 (0.6%)	2 (1.0%)	0 (0.0%)	4 (0.7%)
90–179 days	332 (97.1%)	183 (94.3%)	44 (95.7%)	559 (96.0%)
Over 180 days				
prevalent strains at infection	742 (83.8%)	521 (81.3%)	193 (81.4%)	1,456 (82.6%)
Original strain	1 (0.1%)	0 (0.0%)	0 (0.0%)	1 (0.1%)
Alpha variant	73 (8.2%)	58 (9.0%)	23 (9.7%)	154 (8.7%)
Delta variant	35 (4.0%)	28 (4.4%)	11 (4.6%)	74 (4.2%)
Omicron variant	34 (3.8%)	34 (5.3%)	10 (4.2%)	78 (4.4%)
Number of IgG measurements				
1	93 (10.2%)	14 (2.3%)	0 (0.0%)	107 (6.1%)
2	72 (7.9%)	24 (3.9%)	5 (2.2%)	101 (5.7%)
3	96 (10.5%)	57 (9.2%)	16 (7.0%)	169 (9.6%)
4	181 (19.9%)	124 (19.9%)	65 (28.4%)	370 (21.0%)
5	469 (51.5%)	404 (64.9%)	143 (62.5%)	1016 (57.6%)

COVID-19 during the study period				
No	2264 (64.2%)	2047 (73.5%)	878 (82.6%)	5189 (70.3%)
Yes	1263 (35.8%)	739 (26.5%)	185 (17.4%)	2187 (29.7%)
IgG median (interquartile range)	5784.53(12076.51)	11166.19(18569.08)	18320.54(27151.50)	9426.43(17763.90)

Total of 1763 study participants, with each participant contributing one data point.Data in the table are No. (%).

S_Table 2. Summary of Antibody Titer Data by Previous Infection Status

Number of vaccine doses administered	Months elapsed since vaccination	n	Log-transformed titer		Original antibody titer (AU/ml)		
			Median	Interquartile range	Geometric mean titer	95% Confidence Intervals	
						Lower limit	Upper limit
Never-infected group	Total	6079	3.33	0.96	2115.60	2025.05	2210.21
3	0	59	3.65	1.22	1766.95	684.74	4559.54
	1	68	3.60	0.86	2244.93	1100.46	4579.60
	2	160	3.49	0.70	3065.36	2570.68	3655.23
	3	346	3.17	0.75	1545.31	1264.15	1889.00
	4	634	3.10	0.65	1441.95	1310.55	1586.53
4	5-	1,647	2.91	0.72	907.21	838.20	981.91
	0	589	3.58	1.01	3193.04	2741.34	3719.17
	1	448	3.69	0.70	4604.76	4056.61	5226.98
	2	481	3.55	0.65	3431.40	3022.43	3895.70
	3	362	3.45	0.79	2772.72	2390.87	3215.57
	4	262	3.30	1.02	2001.58	1635.92	2448.99
5	5-	155	3.24	1.04	1567.50	1169.92	2100.18
	0	202	3.78	0.89	5136.87	4209.77	6268.15
	1	288	3.94	0.76	7910.15	6753.15	9265.39
	2	233	3.70	0.75	4279.92	3587.55	5105.91
	3	136	3.72	0.83	4695.93	3708.68	5945.97
	4	9	3.27	0.84	2489.77	920.51	6734.22
Previously-infected group	Total	1297	4.10	0.78	11123.10	10304.61	12006.60
3	0	5	3.94	0.81	7574.08	1988.18	28853.84
	1	12	3.99	0.32	10072.38	5899.01	17198.30
	2	10	3.68	0.68	4206.85	1865.80	9485.24
	3	23	3.72	0.77	4339.16	2376.50	7922.71
	4	37	3.80	1.10	5253.41	3012.11	9162.43
4	5-	526	3.96	0.72	7843.07	6962.21	8835.38
	0	56	4.07	0.90	8929.48	5903.27	13507.00
	1	63	4.21	0.64	16201.38	12363.46	21230.67
	2	78	4.21	0.74	15198.11	11423.47	20220.00
	3	76	4.24	0.72	12151.75	8805.11	16770.37
	4	79	4.20	0.84	15884.32	11784.18	21411.05
5	5-	137	4.27	0.74	16950.92	13754.16	20890.66
	0	28	4.01	0.68	11533.27	6577.27	20223.63
	1	61	4.45	0.64	20532.10	14720.14	28638.81
	2	61	4.43	0.63	22947.20	16930.24	31102.57
	3	42	4.48	0.55	23313.15	16568.62	32803.15
	4	3	4.73	0.85	42715.28	3548.81	514142.70

S_Table 3: Rationale and formal specification of the models

Rationale of the Bayesian models

The goal of the current study was to evaluate whether the dynamics of antibody titers after vaccination differed between individuals with and without prior infection.

Bayesian analysis was used in this study to accommodate the flexibility and complexity of the model in a small sample size and to ensure a stable analysis. The model's flexibility allowed us to account for the random effects of both the intercept and the slope of the antibody titer trajectory over time. We initially attempted a linear mixed effect interval-censored analysis with random intercept and slope, but this did not converge. Bayesian analysis is more flexible than frequentist methods and can handle complex models, so we chose it to ensure that the analysis was stable.

Selection of the models

We selected the Bayesian linear mixed-effect interval-censored model with a random intercept and slope as our primary analysis, aligning with the study goal and data structure. This model provides the flexibility necessary for estimating parameters related to previous infection status, which is a key focus in our research, accommodating upper-censoring above 30,000 AU/mL. By considering both the variability in initial antibody titers (intercept) and the rate of change over time (slope), our model allowed us to explore how previous infection status influences the post-vaccination antibody trajectories, accounting for individual variances. This model appropriately accounts for the nested data structure, incorporating repeated measures within individuals.

A formal specification of the models

All dependent and independent variables and brief model construction are explained in the methods section. The parameter of primary interest is that of previously infected status. The mixed-effect model structure with random intercept and slope is nested within individuals.

We chose an unstructured covariance matrix to allow the model maximum flexibility in capturing the potentially complex relationships between random effects. This flexibility is vital for our data, where repeated measures within individuals may exhibit different patterns that cannot be easily assumed to follow a particular structure. The unstructured matrix allows for a more nuanced and data-driven estimation of the covariance parameters, which is essential for accurately characterizing the variability in both intercepts and slopes across individuals.

Jeffreys priors were chosen for their non-informative nature, providing minimal prior information about the model parameters and allowing the data to primarily drive the posterior distributions. This is particularly important in our study due to the complexity of the model and the small sample size. Jeffreys priors have desirable properties in Bayesian analysis, such as helping to avoid overfitting or underfitting in these scenarios.

We presented the formal specification (computer code) of the models.

<u>Main model</u>

Bayesian linear mixed effect interval-censored model with random intercept and slope

```
bayes, prior({log_result}, jeffreys): metobit log_result days10 i.prior_infection i.prior_infection#c.days10 i.infection_period i.days_since_infection i.prev_vaccine_dose i.prev_vaccine_dose#c.days10 i.age_category i.age_category#c.days10 i.sex i.sex#c.days10 || id: days10, cov(unstructured) ul(4.48)
```

Variables explanation provided below...

log_result: IgG in logarithmic scale
days10: days elapsed after recent vaccination (10 days)
prior_infection: previous COVID-19 infection status
infection_period: major infectious strains at time of infection
days_since_infection: days since recent COVID-19 infection
prev_vaccine_dose: most recent number of vaccine doses
sex: sex
age: age category

Deviance Information Criterion (DIC): 3598.27

Alternative models

Bayesian linear mixed effect interval-censored model with random intercept

bayes, prior({log_result}, jeffreys): metobit log_result days10 i.prior_infection i.prior_infection#c.days10 i.infection_period i.days_since_infection i.prev_vaccine_dose i.prev_vaccine_dose#c.days10 i.age_category i.age_category#c.days10 i.sex i.sex#c.days10 || id:, cov(unstructured) ul(4.48)

Deviance Information Criterion (DIC): 3533.373

Bayesian mixed effect interval-censored model with random intercept and slope including the quadratic term

bayes, prior({log_result}, jeffreys): metobit log_result days10 i.prior_infection i.prior_infection#c.days10 i.infection_period i.days_since_infection i.prev_vaccine_dose i.prev_vaccine_dose#c.days10 i.age_category i.age_category#c.days10 i.sex i.sex#c.days10 || id:, cov(unstructured) ul(4.48)

Deviance Information Criterion (DIC): 3592.071

Bayesian linear random intercept and slope model with replacement of values above 30,000 AU/ml to 30,000

replace log_result=4.48 if log_result>=4.48
bayes, prior({log_result}, jeffreys): mixed log_result days10 i.prior_infection i.prior_infection#c.days10 i.infection_period i.days_since_infection i.prev_vaccine_dose i.prev_vaccine_dose#c.days10 i.age_category i.age_category#c.days10 i.sex i.sex#c.days10 || id: days10, cov(unstructured)

Deviance Information Criterion (DIC): 3154.06

Bayesian linear mixed interval-censored models adjusting for medical history and lifestyle factors on antibody titer trends after vaccination (n=1438 with 6593 data points)

bayes, prior({log_result}, jeffreys): metobit log_result days10 i.prior_infection i.prior_infection#c.days10 i.infection_period i.days_since_infection i.prev_vaccine_dose i.prev_vaccine_dose#c.days10 i.age_category i.age_category#c.days10 i.sex

i.sex#c.days10 i.disease i.disease#c.days10 i.immune i.immune#c.days10 i.smoke i.smoke#c.days10 i.alcohol i.alcohol#c.days10|| id: days10, cov(unstructured) ul(4.48)

Additional variables explanation provided below...

Disease: Underlying medical conditions

Immune: Immunocompromised or on immunosuppressant medication

Smoke: Smoking history

Alcohol: Alcohol consumption history

Deviance Information Criterion (DIC): 3222.74

S_Table 4. Bayesian linear random intercept interval-censored model on antibody titer trends after vaccination

Fixed effects	Posterior median	95% CrI	
(Intercept)	2.764	2.745	2.782
Time (elapsed 10 days)	-0.019	-0.022	-0.015
Previous COVID-19 infection	0.333	0.323	0.344
Previous COVID-19 infection:Time	0.020	0.018	0.022
Post-infection days			
0–6 days	-0.318	-0.322	-0.314
7–89 days	0.361	0.351	0.370
90–179 days	0.274	0.269	0.280
Over 180 days	(omitted)		
prevalent strains at infection			
Alpha variant	0.635	0.623	0.648
Delta variant	0.002	-0.009	0.012
Omicron variant	0.635	0.627	0.643
Recent vaccine dose			
4	0.342	0.326	0.355
5	0.459	0.446	0.474
Recent vaccine dose:Time			
4:Time	0.010	0.005	0.014
5:Time	0.026	0.018	0.035
Age group (years)			
Age 40–59	-0.030	-0.042	-0.018
Age 60–79	-0.111	-0.118	-0.104
Over 80	-0.261	-0.265	-0.258
Age group (years):Time			
Age 40–59:Time	0.000	-0.004	0.003
Age 60–79:Time	0.001	-0.004	0.006
Over 80:Time	-0.003	-0.012	0.004
Female sex	-0.124	-0.135	-0.115
Female sex:Time	0.000	-0.003	0.003

Random-effects parameters (unstructured)	Posterior median	95% CrI	
Variance of random intercept	0.138	0.117	0.162
Variance of the overall error term	0.204	0.190	0.219

References for the categorical variables are as follows:
No history of previous COVID-19 infection is the reference group for the post-infection days category.
Original strain is the reference group for the prevalent strains at infection category.
Dose of 3 is the reference group for the recent vaccine dose category.
Age <40 years is the reference group for the age group category.
Male sex is the reference group for female sex.
Time, post-vaccination days (by 10 days), is a continuous variable from days 0 to 500.
The symbol ":" indicates interaction.
CrI, credible interval

S_Table 5. Main model on antibody titer trends after vaccination (Bayesian linear random intercept and slope interval-censored model) including the quadratic term for time

Fixed effects	Posterior median	95% CrI	
(Intercept)	2.882	2.870	2.896
Time (elapsed 10days): Time (elapsed 10 days)	0.000	0.000	0.000
Time (elapsed 10 days)	-0.031	-0.037	-0.025
Previous COVID-19 infection	0.358	0.345	0.370
Previous COVID-19 infection:Time	0.018	0.015	0.021
Post-infection days			
0–6 days	-0.308	-0.316	-0.299
7–89 days	0.369	0.357	0.382
90–179 days	0.280	0.269	0.291
Over 180 days	(omitted)		
prevalent strains at infection			
Alpha variant	0.639	0.631	0.648
Delta variant	0.010	0.004	0.016
Omicron variant	0.627	0.609	0.646
Recent vaccine dose			
4	0.250	0.241	0.260
5	0.324	0.311	0.337
Recent vaccine dose:Time			
4:Time	0.016	0.012	0.021
5:Time	0.035	0.027	0.044
Age group (years)			
Age 40–59	-0.025	-0.035	-0.015
Age 60–79	-0.109	-0.121	-0.098
Over 80	-0.264	-0.273	-0.253
Age group (years):Time			
Age 40–59:Time	-0.001	-0.005	0.004
Age 60–79:Time	0.004	-0.001	0.008
Over 80:Time	-0.013	-0.023	-0.003
Female sex	-0.121	-0.126	-0.116
Female sex:Time	0.005	-0.003	0.012

Random-effects parameters (unstructured)	Posterior median	95% CrI	
Variance of random intercept	0.189	0.128	0.244
Covariance between random intercept and random slope	-0.008	-0.011	-0.004
Variance of error term for time	0.003	0.003	0.004
Variance of the overall error term	0.189	0.173	0.205

References for the categorical variables are as follows:
No history of previous COVID-19 infection is the reference group for the post-infection days category.
Original strain is the reference group for the prevalent strains at infection category.
Dose of 3 is the reference group for the recent vaccine dose category.
Age <40 years is the reference group for the age group category.
Male sex is the reference group for female sex.
Time, post-vaccination days (by 10 days), is a continuous variable from days 0 to 500.
The symbol ":" indicates interaction.
CrI, credible interval

S_Table 6. Bayesian linear random intercept and slope model with replacement of values above 30,000 AU/ml to 30,000 on antibody titer trends after vaccination

Fixed effects	Posterior median	95% CrI	
(Intercept)	3.000	2.684	3.345
Time (elapsed 10days): Time (elapsed 10 days)	-0.019	-0.032	-0.004
Time (elapsed 10 days)	0.281	0.155	0.403
Previous COVID-19 infection			
Previous COVID-19 infection:Time	0.024	0.019	0.029
Post-infection days			
0–6 days	-0.364	-0.885	0.158
7–89 days	0.238	0.150	0.326
90–179 days	0.185	0.099	0.271
Over 180 days	(omitted)		
prevalent strains at infection			
Alpha variant	0.558	0.134	1.001
Delta variant	-0.285	-0.840	0.269
Omicron variant	0.415	0.097	0.715
Recent vaccine dose			
4	0.309	0.196	0.425
5	0.423	0.251	0.597
Recent vaccine dose:Time			
4:Time	0.007	-0.002	0.016
5:Time	0.013	-0.007	0.033
Age group (years)			
Age 40–59	-0.014	-0.143	0.113
Age 60–79	-0.127	-0.284	0.024
Over 80	-0.432	-0.624	-0.246
Age group (years):Time			
Age 40–59:Time	-0.001	-0.014	0.011
Age 60–79:Time	0.001	-0.014	0.015
Over 80:Time	0.005	-0.016	0.025
Female sex	-0.048	-0.155	0.060
Female sex:Time	-0.003	-0.014	0.009

Random-effects parameters (unstructured)	Posterior median	95% CrI	
Variance of random intercept	0.140	0.104	0.180
Covariance between random intercept and random slope	-0.006	-0.008	-0.003
Variance of error term for time	0.003	0.002	0.003
Variance of the overall error term	0.156	0.145	0.167

References for the categorical variables are as follows:
No history of previous COVID-19 infection is the reference group for the post-infection days category.
Original strain is the reference group for the prevalent strains at infection category.
Dose of 3 is the reference group for the recent vaccine dose category.
Age <40 years is the reference group for the age group category.
Male sex is the reference group for female sex.
Time, post-vaccination days (by 10 days), is a continuous variable from days 0 to 500.
The symbol “:” indicates interaction.
CrI, credible interval

S_Fig. 1. Scatter plot of antibody measurements with overlaid linear regression lines showing temporal changes by prior infection status. Unadjusted (n=1763 with 7372 data points).

The blue line with blue dots represents individuals never infected with COVID -19. The red line with red dots represents the previous ly infected individuals. The dotted line indicates 1000 AU/mL as a reference. Model details, including intercepts, coefficients, and their correspondi ng 95% confidence intervals are provided in the table below the figure. The symbol ":" indicates interaction.

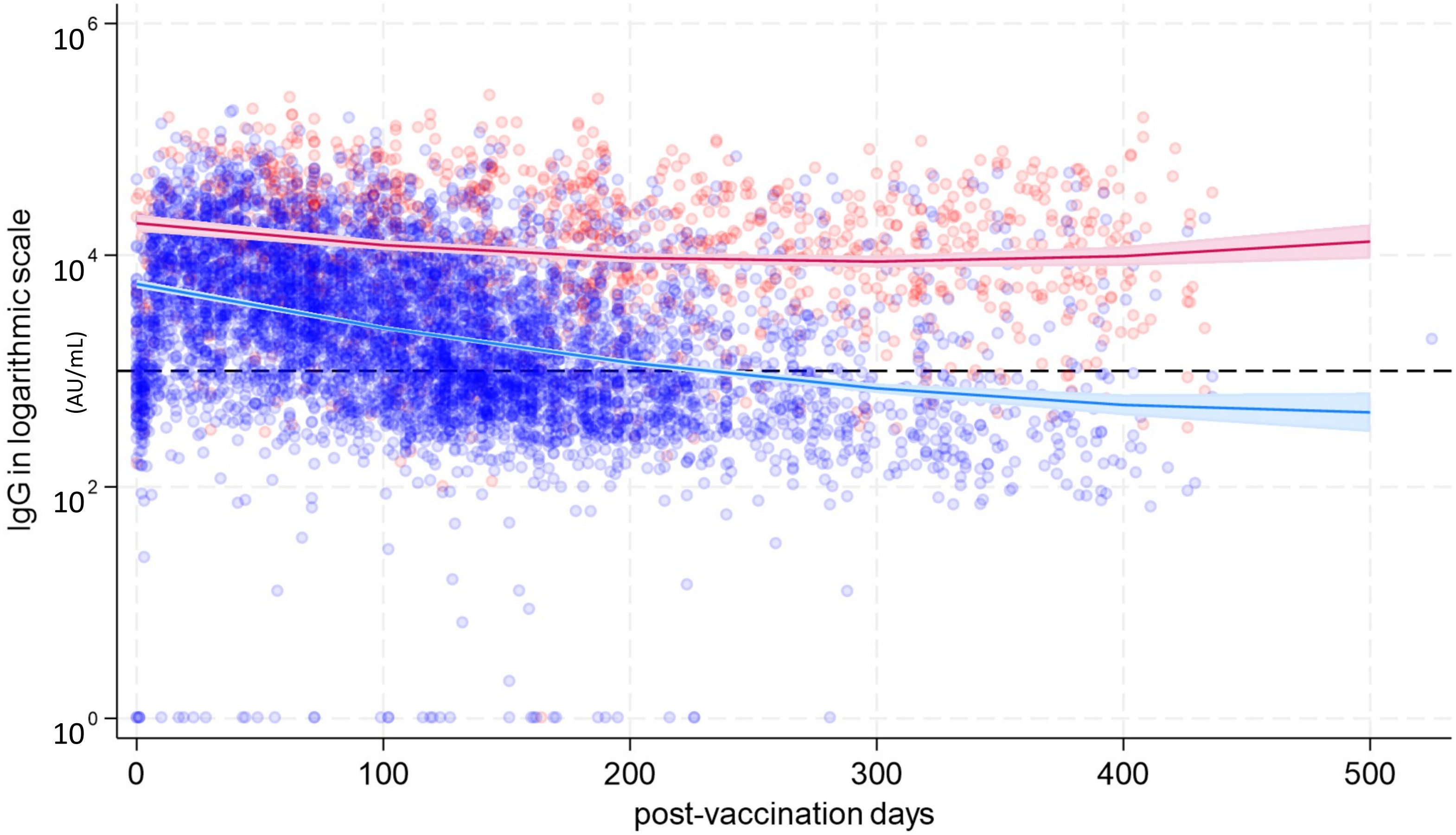

	Coefficient	95% confidence intervals	
(Intercept)	3.674	3.647	3.701
Time:Time	0.000	0.000	0.001
Time (elapsed 10 days)	−0.029	−0.031	−0.027
Previous COVID-19 infection	0.405	0.339	0.472
Time:Previous COVID-19 infection	0.024	0.020	0.027
